# A Study of Small and Large Bowel Wall Thickness Using Computed Tomography and Its Histopathological Correlation

**DOI:** 10.7759/cureus.72932

**Published:** 2024-11-03

**Authors:** Rohit LNU, Sachin Khanduri, Zaara Khan, Danish Ansari, Mohsin Mulani, Ashok Gupta, Nadeem Alam, Akshay Aggarwal, Sana LNU, Aastha Agrawal

**Affiliations:** 1 Radiodiagnosis, Era's Lucknow Medical College and Hospital, Lucknow, IND; 2 Radiology, Era's Lucknow Medical College and Hospital, Lucknow, IND

**Keywords:** bowel wall thickening, ct imaging, diagnostic accuracy, inflammatory bowel disease, neoplastic lesions, small bowel

## Abstract

Background: Small bowel imaging presents significant challenges due to the bowel's length, narrow caliber, and complex looping. Accurate diagnosis of gastrointestinal disorders often requires detailed imaging to differentiate various pathologies, such as inflammatory bowel disease (IBD), infections, ischemic conditions, and neoplasms.

Introduction: The small bowel plays a crucial role in digestion and absorption and is susceptible to various pathologies. CT imaging is essential for diagnosing bowel wall thickening, which can indicate a range of conditions. Dual-energy CT (DECT) and CT enterography offer advanced imaging capabilities to address these diagnostic challenges. This study aims to evaluate the efficacy of CT in staging malignant lesions by correlating imaging findings with histopathology to enhance non-invasive diagnosis and treatment strategies.

Methodology: This cross-sectional study was conducted over two years at Era's Medical College and Hospital, Lucknow, India, with 60 subjects. Patients with abnormal bowel wall thickening (>5 mm) on ultrasound were included, while those with renal dysfunction or pregnancy were excluded. After informed consent, subjects consumed a mannitol solution before undergoing CT scans using a 384-slice Dual Energy CT scanner (Somatom Force, Siemens Healthcare, Erlangen, Germany). All images were post-processed on a workstation using Synovia software (Synovia Solution, Fort Worth, Texas), which allows for image analysis using three-material decomposition. Statistical analysis was performed using IBM SPSS Statistics for Windows, Version 26 (Released 2019; IBM Corp., Armonk, New York).

Results: The majority of patients were young adults aged 20-39 years (63.33%), with a slight male predominance (53.33%). Abdominal pain was the most common complaint (35.00%). Mild wall thickening (<10 mm) was associated with IBD (48.28%), while marked thickening (>10 mm) was linked to neoplastic lesions (48.39%). Symmetrical thickening was common in infective and inflammatory conditions, whereas asymmetrical thickening was typical of neoplastic lesions. CT scans demonstrated high diagnostic accuracy, with 83.33% sensitivity, 95.24% specificity, 88.24% positive predictive value, and 93.02% negative predictive value, resulting in an overall accuracy of 91.67%.

Conclusion: The study highlights that neoplastic lesions are associated with marked bowel wall thickening, while inflammatory conditions present with mild thickening. CT scans proved highly effective in diagnosing gastrointestinal disorders, with significant accuracy in distinguishing between benign and malignant lesions. This underscores the importance of advanced imaging techniques in clinical practice for improved patient outcomes.

## Introduction

The small bowel, integral to digestion and absorption, presents significant challenges in imaging due to its length, narrow caliber, and complex looping [1]. It plays a crucial role in digestion, absorption, and protection against harmful substances and bacteria. This intricate anatomy, along with distinctive vascular patterns and physiological functions, makes the small bowel more susceptible to various pathological conditions compared to the large bowel [2,3].

In diagnosing colorectal lesions, CT imaging frequently identifies colon wall thickening, which has a broad differential diagnosis [4]. Key CT features for evaluating wall thickening include the degree of thickening, symmetry, contour irregularities, and enhancement patterns [5,6]. Additional factors, such as exophytic components, lymphadenopathy, and adjacent inflammatory responses, are crucial for differential diagnosis. Dual-energy computed tomography (DECT) enhances imaging by providing material-specific imagery through low- and high-energy attenuation data [7,8].

Acute small bowel issues often present as diffuse abdominal pain, requiring imaging to determine the underlying cause. Intravenous contrast-enhanced CT is the preferred method for assessing acute small bowel conditions, distinguishing them from other causes of acute abdomen [9]. Multidetector CT offers high spatial and contrast resolution, aiding in the diagnosis of mesenteric ischemia, inflammatory bowel disease (IBD), and small bowel obstructions. CT enterography is especially valuable for evaluating IBD and identifying complications, while routine CT scans are used for other small bowel pathologies [10].

Technical challenges in small bowel imaging include its long, serpentine structure and motion artifacts from peristalsis and breathing. Traditional methods, such as small bowel follow-through and enteroclysis, provide indirect information and may be limited by complications from overlapping loops. While CT enteroclysis offers detailed imaging, it is invasive and costly. Enteroscopy, although promising, cannot visualize the entire small intestine [11-14].

CT enterography, introduced by Raptopoulos et al. in 1997, addresses these challenges by allowing assessment of individual intestinal segments without loop superimposition. It provides comprehensive insights into the bowel wall, mesentery, and surrounding structures. Neutral contrast agents, which have attenuation values similar to water, are now preferred in CT enterography as they avoid masking mucosal enhancement and improve visualization of mesenteric vessels. CT enterography is valuable in diagnosing a range of conditions, including IBDs, gastrointestinal bleeding, and neoplasms [15].

This study aims to evaluate the efficacy of CT in staging malignant lesions by correlating imaging findings with histopathology. Understanding this correlation can enhance non-invasive diagnosis, improve treatment strategies, and advance patient care in gastroenterology.

## Materials and methods

This cross-sectional study was conducted over two years at the Departments of Surgery and Radiodiagnosis at Era's Medical College and Hospital, Lucknow, India (IRB Approval: ELMC&H/R.Cell 12023/115; dated December 28, 2023), with a sample size of 60 subjects. The sample size was calculated based on the variation in mean bowel wall thickness between two study groups: malignant and non-malignant. The standard deviations for the malignant and non-malignant groups were 15.68 and 3.69, respectively. With a type I error (α) of 5% and a type II error (β) of 20%, the study was designed to achieve 90% power, resulting in a required sample size of 30 subjects per group.

Subjects included in the study had abnormal bowel wall thickening (>5 mm) detected on ultrasound. Patients with impaired renal function or who were pregnant were excluded. Following ultrasound screening, eligible subjects were enrolled after informed consent was obtained. Demographic data were recorded, and each subject was instructed to consume 1.5 to 2.0 liters of a 3% mannitol solution before undergoing a CT scan. The scans were conducted using a 384-slice Dual Energy CT scanner (Somaton Force, Siemens Healthcare, Erlangen, Germany), and all images were post-processed on a workstation using Synovia software (Synovia Solution, Fort Worth, Texas), allowing analysis using three-material decomposition. Before the injection of an intravenous contrast agent, a region of interest (ROI) was marked over the abdominal area. A bolus of iso-osmolar non-ionic iodinated contrast (Iohexol 350 mg/mL) was administered at 1.5-2 mL per kg into the antecubital vein. The study aimed to evaluate bowel wall pathologies, with statistical analysis conducted to reach final conclusions based on the findings.

Statistical analysis

IBM SPSS Statistics for Windows, Version 26 (Released 2019; IBM Corp., Armonk, New York) was used for statistical analysis. Categorical variables were expressed as frequencies and percentages. Nominal categorical data between groups were compared using the chi-squared test or Fisher’s exact test, depending on data characteristics. Diagnostic test values such as sensitivity, specificity, positive predictive value (PPV), and negative predictive value (NPV) were calculated to assess the accuracy of CT in detecting malignant and benign bowel lesions. A p-value of less than 0.05 was considered statistically significant for all tests.

## Results

The study involved a detailed analysis of the enrolled patients, focusing on various demographic and clinical parameters. Age distribution data revealed that the majority of patients (33.33%) were in the 20-29 years age group, followed closely by 30.00% in the 30-39 years group. The study population also included 20.00% of patients aged 40-49 years, while the smallest groups were those aged 18-20 years (3.33%) and 50-59 years (13.33%). Regarding gender, the study showed a slight male predominance, with 53.33% of patients being male and 46.67% female (Table 1).

**Table 1:** Sociodemographic distribution of enrolled patients.

Sociodemographic parameters	Category	N=60	%
Age (years)	18-20	2	3.33
	20-29	20	33.33
	30-39	18	30.00
	40-49	12	20.00
	50-59	8	13.33
Gender	Male	32	53.33
	Female	28	46.67

**Figure 1:**
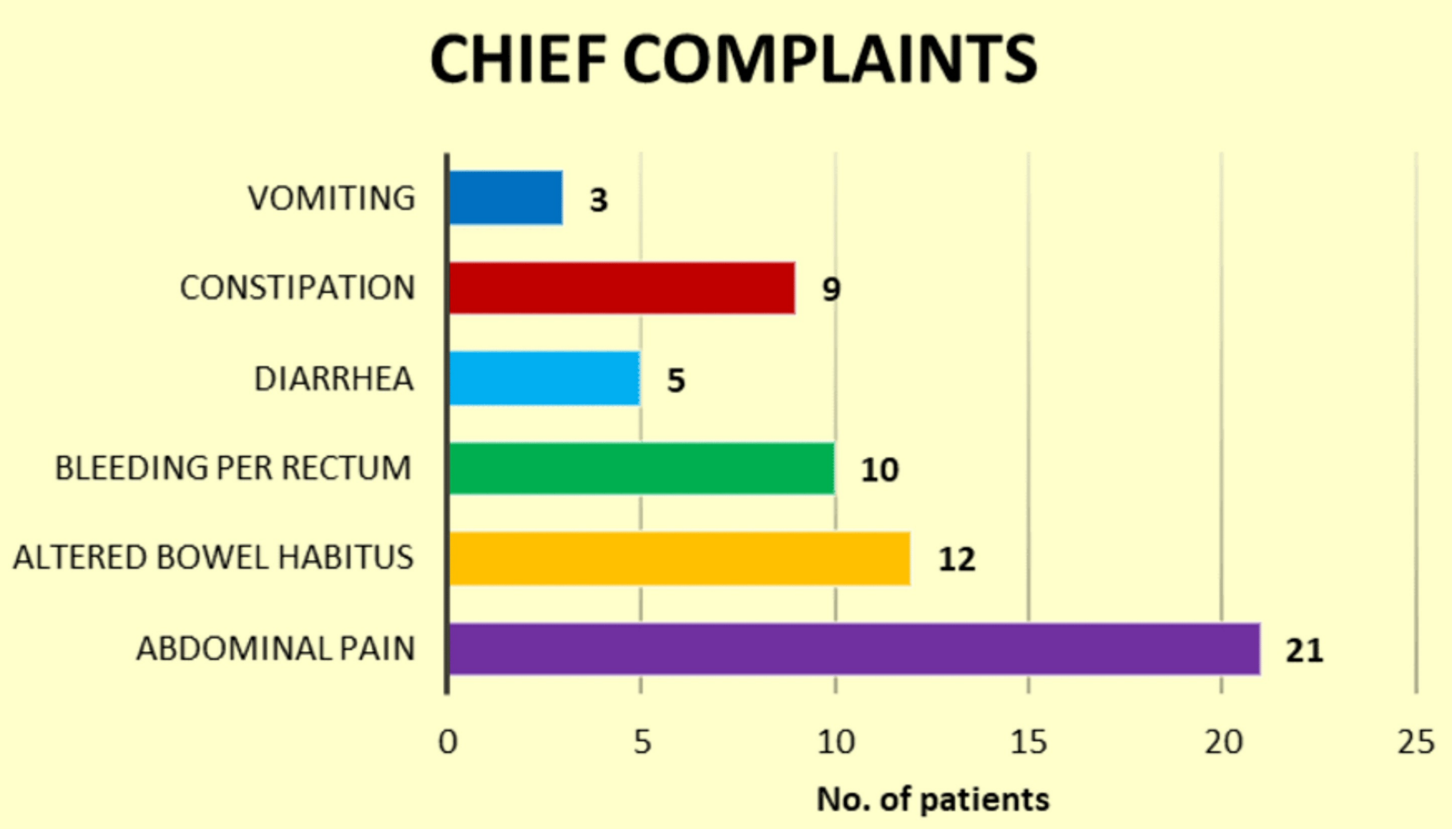
Graphical representation of the chief complaints of the enrolled patients.

Figure 1 depicts the chief complaints of the enrolled patients, highlighting the most common symptoms they presented with. Abdominal pain was the leading complaint, affecting 35% of participants, followed by altered bowel habits (20%), bleeding per rectum (16.67%), constipation (15%), diarrhea (8.33%), and vomiting (5%). This graphical representation visualizes the range and frequency of symptoms associated with gastrointestinal conditions, providing a clear summary of patient experiences.

**Figure 2:**
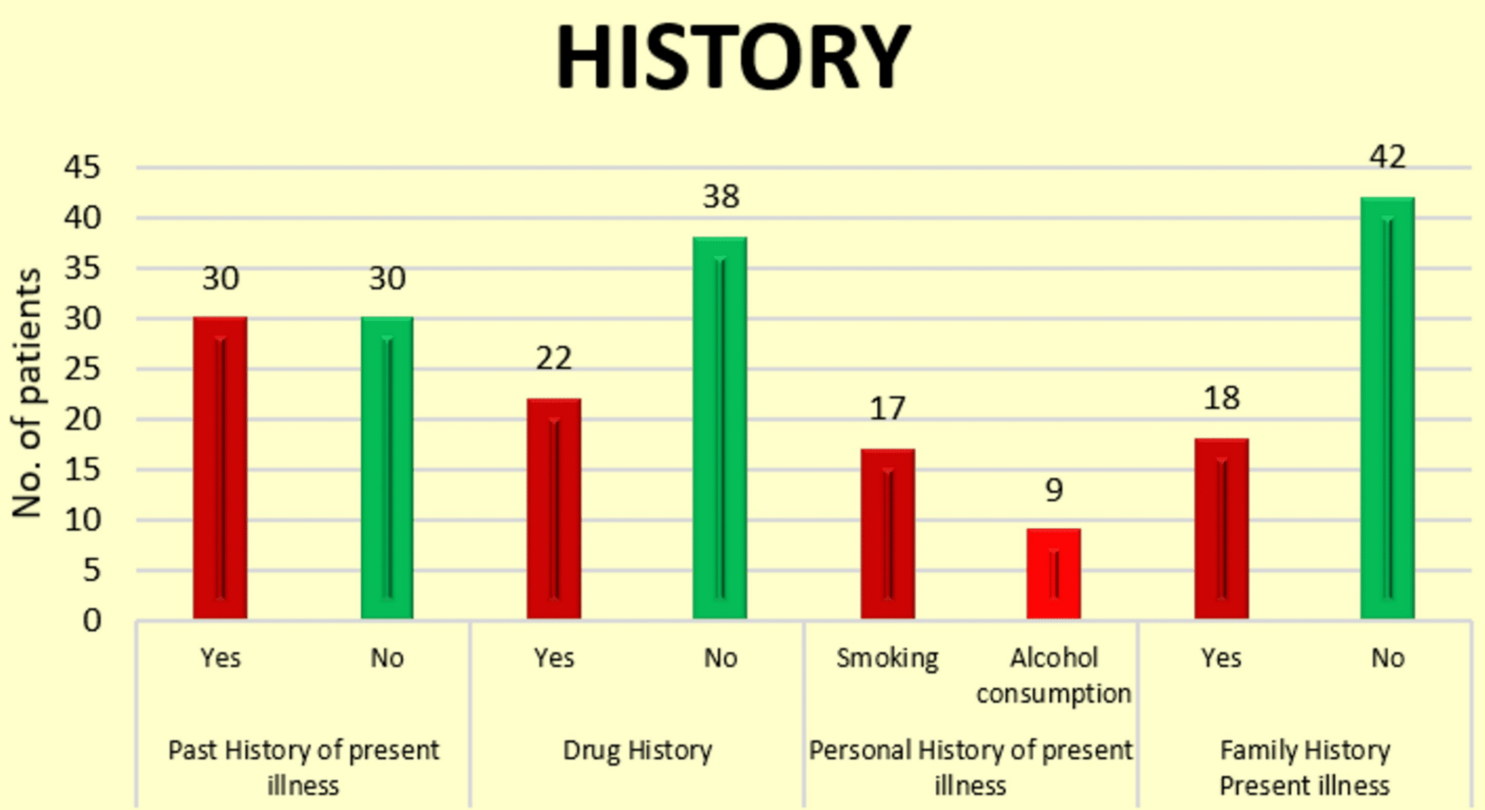
Graphical representation of the various history of the enrolled patients.

Figure 2 illustrates patient history, focusing on personal and family health details. It categorizes patients based on their history of smoking, alcohol consumption, drug use, personal history of the present illness, and family history. Notably, 50% of patients had a history of the present illness, 36.7% reported drug use, and 30% had a family history of similar conditions. Including data on smoking and alcohol consumption provides additional context for potential lifestyle-related factors influencing patient health outcomes. These figures visually complement the text, reinforcing the study's focus on the demographic and clinical characteristics of gastrointestinal disorder patients, particularly regarding common complaints and relevant medical histories.

Regarding bowel wall pathologies, the study categorized patients based on wall thickness, symmetry, length of involvement, and enhancement patterns. Among those with mild wall thickening (<10 mm), IBD was most common (48.28%), while neoplastic lesions were predominant (48.39%) in those with marked thickening (>10 mm). The distribution of bowel wall pathologies was statistically significant (p=0.0002) among the Wall thickness (mild and marked) (Table 2).

**Table 2:** Bowel wall pathologies according to the thickness of the bowel wall of the enrolled patients. ^*^Chi-square test. Significant at p<0.001.

Bowel wall pathologies	Wall thickness (in mm)		P-value
	Mild (<10 mm), N (%)	Marked (>10 mm), N (%)	
Infective (TB)	13 (44.83)	3 (9.68)	X=19.81; p = 0.0002*
Inflammatory (IBD)	14 (48.28)	10 (32.26)	
Ischemic (ISC)	0 (0.00)	3 (9.68)	
Neoplastic lesions of the bowel	2 (6.90)	15 (48.39)	
Total	29 (100)	31 (100)	

Symmetrical thickening was more common in infective and inflammatory conditions, whereas neoplastic lesions were more likely to cause asymmetrical thickening (54.17%). In terms of focal versus segmental and diffuse thickening, neoplastic lesions were primarily focal, while IBD was more commonly associated with segmental and diffuse thickening. The distribution of bowel wall pathologies was statistically significant (p<0.0001) in relation to the symmetry of wall thickening (Table 3).

**Table 3:** Symmetry of wall thickening of the enrolled patients. ^*^Chi-square test. Significant at p<0.001.

Symmetry of wall thickening	Symmetrical, N (%)	Asymmetrical, N (%)	P-value
Infective (TB)	14 (38.89)	2 (8.33)	X=16.53; p=0.0009*
Inflammatory (IBD)	15 (41.67)	9 (37.50)	
Ischemic (ISC)	3 (8.33)	0 (0.00)	
Neoplastic lesions of the bowel	4 (11.11)	13 (54.17)	
Total	36 (100)	24 (100)	

The enhancement patterns revealed that homogeneous enhancement was typical of IBD (55.26%), whereas heterogeneous enhancement was more common in neoplastic lesions (68.18%). The distribution of bowel wall pathologies was statistically significant among the enhancement patterns of thickened bowel walls (homogeneous and heterogeneous) (Table 4).

**Table 4:** Enhancing patterns of thickened bowel wall according to the contrast uptake of the enrolled patients. ^*^Chi-square test. Significant at p<0.0001.

Enhancing patterns of the thickened bowel wall	Homogeneous, N (%)	Heterogeneous, N (%)	P-value
Infective (TB)	12 (31.58)	4 (18.18)	X=28.18; p<0.0001*
Inflammatory (IBD)	21 (55.26)	3 (13.64)	
Ischemic (ISC)	3 (7.89)	0 (0.00)	
Neoplastic lesions of the bowel	2 (5.26)	15 (68.18)	
Total	38 (100)	22 (100)	

The study also assessed the diagnostic accuracy of CT scans in identifying malignant and benign lesions. Of the 17 cases identified as malignant by CT, 15 were true positives, resulting in a sensitivity of 83.33% and a specificity of 95.24%. The PPV was 88.24%, while the NPV was 93.02%. Overall, the CT scans demonstrated an accuracy of 91.67%, indicating a significantly high level of reliability (p<0.0001) in diagnosing bowel wall lesions (Table 5). This comprehensive analysis provides valuable insights into the clinical and demographic characteristics of patients with bowel wall pathologies, enhancing our understanding of these conditions and their diagnosis.

**Table 5:** CT Imaging in differentiating malignant and benign lesions compared to histopathology. *Chi-square test. Significant at p<0.0001. PPV: positive predictive value, NPV: negative predictive value.

	Malignant lesions (histopathology)	Benign lesions (histopathology)	Total (CT)
Malignant (CT)	15	2	17
Benign (CT)	3	40	43
Total (histopathology)	18	42	60
Diagnostic test value			
Sensitivity	83.33%	PPV	88.24%
Specificity	95.24%	NPV	93.02%
Accuracy	91.67%	p-value	<0.0001*

**Figure 3:**
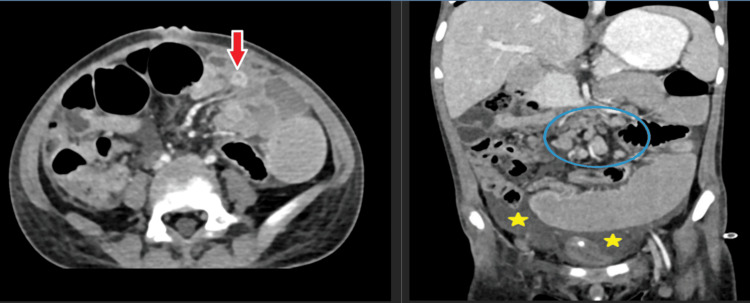
Abdominal tuberculosis with small bowel obstruction (case 1). Contrast-enhanced computed tomography (CECT) abdomen axial section shows multiple dilated small bowel loops due to strictures (red arrows). The coronal section displays enlarged mesenteric lymph nodes (blue circle) and ascites (yellow stars).

**Figure 4:**
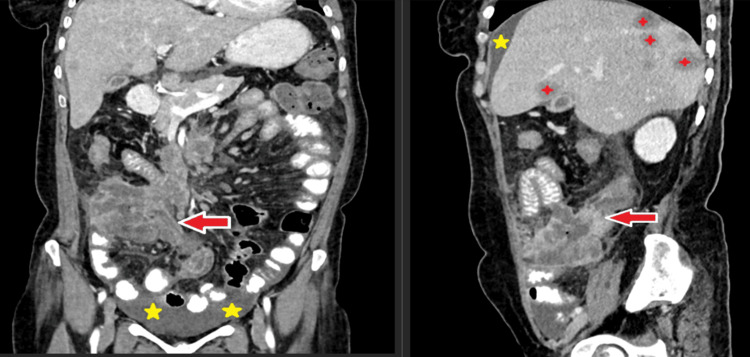
Malignant thickening of bowel wall with secondary hepatic and lymph nodal metastasis and ascites (case 2). Contrast-enhanced computed tomography (CECT) abdomen coronal and sagittal sections show marked heterogeneous enhancing thickening of the distal ileum, ileocecal junction, and part of the cecum, with loss of bowel wall layer definition (red arrows) involving a segmental length, along with secondary ascites (five-point yellow stars) and hepatic metastasis (four-point red stars).

This comprehensive analysis provides valuable insights into the clinical and demographic characteristics of patients with bowel wall pathologies, enhancing understanding of these conditions and their diagnosis. The axial section of the contrast-enhanced computed tomography (CECT) scan for case 1 (Figure 3) demonstrates multiple dilated small bowel loops, indicated by red arrows, suggesting the presence of strictures likely contributing to the observed obstruction. In the coronal section, enlarged mesenteric lymph nodes, circled in blue, may indicate underlying inflammation or infection. Additionally, ascites is clearly noted, marked with yellow stars, suggesting fluid accumulation in the peritoneal cavity. These findings are consistent with a pathological process affecting the small bowel and adjacent mesentery. In this case, the patient was diagnosed with abdominal tuberculosis, which presented as small bowel obstruction. The imaging findings correlate with typical manifestations of abdominal tuberculosis, including lymphadenopathy, bowel strictures, and ascites. The coronal and sagittal sections of the CECT scan for case 2 (Figure 4), in a diagnosed case of malignant neoplasm of the bowel, show marked heterogeneous enhancing thickening of the distal ileum, ileocecal junction, and a portion of the cecum, as indicated by red arrows. This thickening is associated with a loss of the normal stratification of the bowel wall layers, consistent with neoplastic tissue involvement. The affected segment extends over a considerable length of the bowel. Secondary ascites, marked by five-point yellow stars, suggests fluid accumulation related to the underlying malignancy. Hepatic metastasis is clearly identified, denoted by four-point red stars, indicating the spread of the malignant neoplasm to the liver. These findings, along with the presence of mesenteric lymph node metastasis, indicate advanced-stage disease with hepatic and nodal involvement.

## Discussion

Bowel wall thickening is a key radiological finding in gastrointestinal imaging, indicating various conditions such as IBD, infections such as tuberculosis, ischemic disorders, and neoplastic lesions. Accurate diagnosis requires understanding the patterns of thickening, underscoring the importance of imaging in gastroenterology. Following contrast injection, the intestinal mucosa, the most vascularized layer, becomes more distinct on imaging. Although the submucosa is usually less visible due to lower vascularization, it can be detected when affected by edema, hemorrhage, or fat infiltration [16,17]. On CT scans, bowel wall enhancement should be classified in the "white attenuation pattern" if it matches the venous opacification seen in the same scan [18]. According to Fernandes et al. [16], specific bowel wall attenuation patterns are linked to various clinical conditions. The "gray attenuation pattern," characterized by a thickened gut wall with uniform enhancement resembling muscle, is seen in ischemia and IBD. The "black attenuation pattern," associated with ischemia, infection, and trauma, indicates pneumatosis. The "water halo sign," observed in radiation damage, vascular issues, infections, and idiopathic IBD, describes a stratified, symmetrically thickened bowel wall. The "fat halo sign," a three-layered target with fatty attenuation in the submucosal layer, is common in ulcerative colitis and Crohn's disease. This pattern also appears in rare conditions like chronic radiation enteritis and exposure to cytoreductive therapy [19].

In our study, most patients were in the younger to middle-aged brackets, with 33.33% aged 20 to 29 years and 30.00% aged 30 to 39 years. The 40 to 49 years age group comprised 20.00% of patients, while 13.33% were aged 50 to 59 years. The smallest group was aged 18 to 20 years (3.33%). Overall, 63.33% of patients were between 20 and 39 years, highlighting a demographic concentration in this age range. In the study by Poonam et al. [20], the average age of the participants was 38.24 ± 19.03 years. Our findings are consistent with those observed by Bassiouny et al. [21]. However, our results contrast with those of Poonam et al. [20], who reported a majority of participants (40%) in the age group of 41 to 60 years, followed by 30% in the 21 to 40 years age group, and 24% below 20 years of age. Additionally, Sheikh et al. [22] noted a lower proportion (8.3%) of participants below 20 years of age in their study. These variations highlight different age demographics across various studies, indicating potential regional or demographic differences in the prevalence of the condition under investigation.

In our study, 53.33% of patients were male and 46.67% were female, showing a slight male predominance. This gender distribution is consistent with trends in other clinical studies, where gender differences in disease prevalence or healthcare-seeking behaviors can impact demographics and outcomes. In the Poonam et al. [20] study, 40% of the participants were female and 60% were male. Sheikh et al.'s [22] findings align with the male predilection seen in Poonam et al.'s [20] study, as does Bassiouny et al.'s [21] study, indicating comparable gender distributions across these studies. These consistent patterns underscore the importance of considering gender demographics in clinical research and healthcare planning.

In our study, abdominal pain was the most common complaint, affecting 35.00% of participants, followed by altered bowel habits (20.00%) and bleeding per rectum (16.67%). Constipation, diarrhea, and vomiting were also reported. These findings highlight the diverse clinical presentations of gastrointestinal disorders, with abdominal pain being the most prevalent symptom requiring careful evaluation. In contrast to our study, where abdominal pain was the most prevalent complaint (35.00%), Poonam et al. [20] and Bassiouny et al. [21] reported a 100% incidence of abdominal pain among their participants. However, Poonam et al. [20] observed higher frequencies of symptoms like weight loss (46%), fever (44%), vomiting (42%), and diarrhea/constipation (22%). In contrast, Bassiouny et al. [21] reported fever (15%), constipation (24%), diarrhea (26%), and vomiting (41%). These differences highlight the varied clinical presentations of gastrointestinal disorders across studies, influenced by regional and demographic factors.

Our study shows distinct trends in bowel wall thickening patterns among different pathologies. Symmetrical thickening is common in infective (38.89%) and inflammatory (41.67%) conditions, while asymmetrical thickening is less frequent in these (8.33% and 37.50%, respectively). Ischemic causes exclusively present symmetrical thickening (8.33%), whereas neoplastic lesions mainly show asymmetrical thickening (54.17%). These patterns are crucial for the accurate diagnosis and management of bowel wall pathologies. Similarly, Insko et al. [23] reported that 71% of malignant cases exhibited asymmetrical thickening, while 29% showed symmetrical thickening. For benign cases, 25% had asymmetrical and 75% had symmetrical thickening, compared to 33% and 67% in our study. This indicates a slightly higher prevalence of asymmetrical thickening in malignant cases and symmetrical thickening in benign cases in our findings. Similarly, our study supports the effectiveness of CT as a primary tool for evaluating bowel wall thickening.

In our study, homogeneous enhancement of the thickened bowel wall was most commonly seen in IBD (55.26%), followed by tuberculosis (31.58%), ischemic pathology (7.89%), and neoplastic lesions (5.26%). In contrast, heterogeneous enhancement was primarily linked to neoplastic lesions (68.18%), with tuberculosis and IBD accounting for 18.18% and 13.64% of cases, respectively. No ischemic cases showed heterogeneous enhancement. These distinct enhancement patterns are crucial for differentiating bowel pathologies in imaging diagnostics. In 36% of instances in our investigation, the primary wall enhancement pattern was homogeneous, which is consistent with the findings of Bhalothia et al. [24] and Megally et al. [25], where mucosa and submucosa were found to be impacted in 38% and 4% of cases, respectively. According to Tapasvi et al. [26], heterogeneous enhancement is a significant marker for malignant lesions, with 20 out of 23 cases showing malignant intestinal wall thickening. Additional indicators of malignancy include asymmetrical thickening and gray attenuation. The study also found CT to have high sensitivity (97%) and specificity (93%) in distinguishing benign from malignant bowel wall thickening. This finding was in line with that of Insko et al.'s research.

In our study, neoplastic lesions were most common in focal thickening (76.19%), while TB and IBD were less frequent. Segmental thickening was mainly associated with IBD (56.25%), and diffuse thickening was predominantly linked to IBD (71.43%). These patterns aid in distinguishing between different bowel wall pathologies for better diagnosis and management. According to Megally et al. [25], the layers that were most frequently affected were the mucosa and/or the submucosa. Conversely, Bhalothia et al. [24] reported that in their investigation, 50% of cases were segmental. The study by Poonam et al. [20] demonstrated a similar percentage of small and large bowel involvement. Tapasvi et al. [26] found that focal and segmental bowel wall involvement were strong indicators of malignancy, with gray attenuation patterns also frequently associated with malignant cases. Our study aligns with these findings, showing similar patterns of bowel involvement and thickening, supporting their diagnostic significance.

In our study, 50% of patients had a past history of present illness, 36.7% reported drug use, 28.3% were smokers, 15.0% consumed alcohol, and 30.0% had a family history of the illness. These findings highlight significant health and lifestyle factors. In contrast, Finkelstone et al. [27] provided broader diagnostic categories without detailed quantitative data or pathological confirmations, which may impact diagnostic specificity.

Mild wall thickening (<10 mm) in our study was mainly associated with IBD (48.28%) and infective pathologies (44.83%), while marked thickening (>10 mm) was most commonly linked to neoplastic lesions (48.39%). This suggests that severe thickening is often indicative of neoplastic lesions, whereas mild thickening is more related to inflammatory and infective conditions. Clinical, histological, and surgical methods confirmed 82% of tuberculosis cases and 18% of cancer cases in our study. We observed varied gastrointestinal pathologies, including large bowel obstruction (10%), mild intestinal obstruction (12%), Crohn’s disease (8%), appendicitis (8%), diverticulitis (6%), and other conditions. Concordance between final and MDCT diagnoses was 88%. Poonam et al. [20] noted different frequencies of mural thickness, with necrotic and non-necrotic nodes found in 42% and 38% of cases, respectively. Bhalothia et al. [24] observed that moderate and mild wall thickening was common in non-malignant conditions, while severe thickening was more indicative of malignancy. Megally et al. [25] found enlarged mesenteric lymph nodes in 31.8% of cases.

In our study, CT scans exhibited strong diagnostic performance for detecting gallbladder lesions, with a sensitivity of 83.33%, specificity of 95.24%, PPV of 88.24%, and NPV of 93.02%, resulting in an overall accuracy of 91.67%. Mangini et al. [28] reported 82.4% concordance between MDCT findings and discharge diagnoses for acute bowel disease, while Bassiouny et al. [21] found an overall sensitivity of 90%, specificity of 93%, PPV of 98%, and NPV of 71%. Ramaswamy et al. [29] achieved an 88.3% accuracy rate for small intestinal pathologies, and Jyothi et al. [30] accurately identified all 26 malignant lesions. Bas et al. [31] demonstrated high sensitivity (95.6%) and specificity (90.4%) for malignant disease, with even higher values for identifying IBD at 97.2% sensitivity and 97.9% specificity. In contrast, Colvin et al. [32] reported 100% sensitivity and 95.7% specificity for colon cancer. Studies by Stermer et al. [33] and Nicholson et al. [34] provided additional insights into diagnostic accuracy for wall thickening and pathology detection, while Horsthuis et al. [35] found high success rates for IBD detection. Although our study did not perform statistical analysis for diverticulitis due to its minor representation, existing literature, including meta-analyses and research by Andeweg et al. [36] and Sallinen et al. [37], highlighted varied detection rates and the need for standardized diagnostic protocols to enhance the effectiveness of MDCT across diverse gastrointestinal conditions.

The analysis of patient demographics and clinical outcomes highlights the importance of detailed medical research. A concentration of cases was observed in young to middle-aged adults, with a slight male predominance. Chief complaints and medical histories revealed prevalent symptoms and associated factors. Diagnostic parameters, including bowel wall pathology and enhancement patterns, provided detailed disease characterization. The high diagnostic accuracy of CT scans, demonstrated by their sensitivity, specificity, and predictive values, underscores their crucial role in clinical practice. Overall, this comprehensive study emphasizes the value of meticulous research in advancing medical knowledge and improving patient care.

Strengths

The study offers specific imaging criteria to differentiate various gastrointestinal pathologies, improving diagnostic accuracy. Strong sensitivity and specificity results validate CT as a reliable tool for evaluating bowel wall thickening. Additionally, the analysis of demographic factors such as age, gender, and lifestyle provides context for disease trends, supporting more targeted healthcare planning.

Limitations

The study’s concentration on younger and middle-aged patients reduces its generalizability, potentially overlooking variations in older or more diverse populations. The small sample size restricts statistical analysis for less common conditions like diverticulitis, limiting insights into these specific pathologies. Furthermore, the study’s cross-sectional design limits the ability to understand disease progression over time.

## Conclusions

This study examined the demographic and clinical characteristics of patients with gastrointestinal disorders, primarily adults aged 20 to 49 years, with a slight male majority (53.33%). Abdominal pain was the most frequent symptom, followed by altered bowel habits and bleeding per rectum. Notably, neoplastic lesions were associated with marked bowel wall thickening (>10 mm), while mild thickening (<10 mm) was linked to IBD. Symmetrical thickening was common in infective and inflammatory conditions, whereas asymmetrical thickening was typical of neoplastic lesions. CT scans demonstrated high accuracy in diagnosing these conditions, with 83.33% sensitivity and 95.24% specificity. The findings underscore the importance of comprehensive clinical evaluation and advanced imaging techniques in diagnosing and managing gastrointestinal disorders, contributing to improved patient outcomes.
